# Feed preference in lactating dairy cows for different pellet formulations

**DOI:** 10.3168/jdsc.2023-0517

**Published:** 2024-02-29

**Authors:** A.L. Carroll, G.M. Fincham, K.K. Buse, P.J. Kononoff

**Affiliations:** Department of Animal Science, University of Nebraska–Lincoln, Lincoln, NE 68503

## Abstract

Two taste preference experiments were conducted with the same 8 multiparous lactating Jersey cattle (100 ± 7.1 DIM, 30.5 ± 4 0.06 kg of milk yield, 18.8 ± 2.52 kg of DMI in experiment 1; and 215 ± 7.1 DIM, 27.6 ± 3.98 kg of milk yield, 19.6 ± 3.03 kg of DMI in experiment 2). In experiment 1, 4 pellets were formulated and manufactured into 4.0-mm pellets. These were as follows: 45.7% alfalfa meal, 45.7% corn grain, and 8.57% wheat middlings (ALFC); 72.3% corn grain, 18.5% wheat middlings, and 9.25% dried distillers grains and solubles (ENG); a pellet containing 100% dehydrated alfalfa meal (DALF); and a pellet containing a mixture of concentrate ingredients (GMIX; 43.1% corn grain, 26.3% dried distillers grains and solubles, 13.8% wheat middlings, 7.10% dry molasses, 3.18% soybean meal, 0.93% corn oil, and 5.6% minor constituents). Cows were offered 0.50 kg of pellets in a randomized arrangement within the feed bunk. Feed preference was ranked from 1 to 4 with 1 being the most preferred and 4 the least. The resulting preference rankings were averaged (± SE) resulting in a highest (closest to 1) to lowest (furthest from 1) ranking as follows of ALFC (1.38 ± 0.164), ENG (2.13 ± 0.327), GMIX (2.88 ± 0.375), and DALF (3.13 ± 0.350). The probabilities of first choice were 70.6 ± 0.55% ALFC, 16.5 ± 0.46% ENG, 5.50 ± 0.475% DALF, and 7.48 ± 0.455% GMIX. A *Z*-test was conducted to determine the percentage a treatment would be chosen first differed from the value of no preference at 25%; ALFC and DALF differed from the mean value, whereas no difference was observed for ENG and GMIX. The most preferred pellet (ALFC) was used in a second study and compared against 3 other treatments in which different flavoring agents were added. In this study, 4 pellets were manufactured with ALFC: 45.7% alfalfa meal, 45.7% corn grain, 6.76% wheat middlings, and 1.81% oregano leaf (ALFCO); 45.7% alfalfa meal, 45.7% corn grain, 8.22% wheat middlings, 0.10% melon flavoring, and 0.25% BitterOff (ALFCM); and 45.7% alfalfa meal, 45.7% corn grain, 8.47% wheat middlings, and 0.10% licorice flavoring (ALFCL). The resulting preference rankings were averaged resulting in a highest to lowest ranking as follows: ALFC (1.25 ± 0.164), ALFCO (2.38 ± 0.263), ALFCM (2.63 ± 0.375), and ALFCL (3.25 ± 0.164). The probabilities of first choice were 81.9 ± 0.65% ALFC, 8.49 ± 0.46% ALFCO, 6.50 ± 0.481% ALFCM, and 3.12 ± 0.491% ALFCL. Of the pellet choices, ALFC and ALFCL differed from the mean value of no choice, whereas no difference was observed for ALFCO and ALFCM. Mixtures of corn grain and dehydrated alfalfa meal bound by wheat middlings may serve as a feeding strategy that is preferred by the animals and may be an effective reward to cows entering an automated milk system, and we were unable to improve preference by adding flavoring agents.

## Highlights

• We examined feed preference of 4 pellet formulation strategies and 3 flavoring agents.
• Pellets were corn and alfalfa mix, dehydrated alfalfa meal, concentrate mix, and high energy.
• Flavorings were melon + a bitterness taste suppressor, oregano, and licorice.
• Animals preferred alfalfa and corn mix even when flavoring agents were added.

## Graphical Abstract

**Figure:**
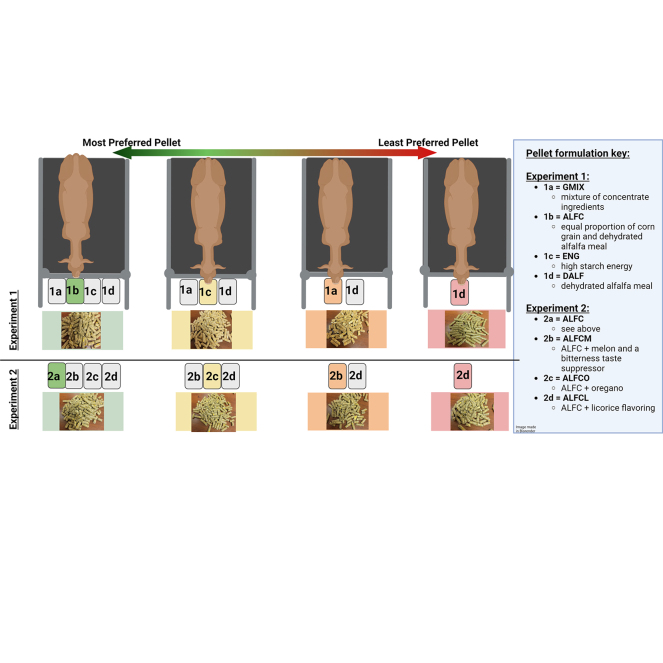
**Summary:** Two experiments were conducted to examine different pellet formulations on feed preference of lactating Jersey cattle. In the first experiment, 4 pelleting formulations were compared: a pellet containing an equal proportion of alfalfa meal and corn grain (ALFC), a high starch energy pellet (ENG), a pellet containing dehydrated alfalfa meal (DALF), and a pellet containing a mixture of concentrate ingredients (GMIX). In the second experiment, 3 flavoring agents, including oregano (ALFCO), licorice (ALFCL), and melon + a bitterness taste suppressor (ALFCM), were added to the most preferred pellet observed in experiment 1 (ALFC). Feed preference rankings from greatest to least preferred were ALFC, ENG, GMIX, and DALF in experiment 1, and ALFC, ALFCO, ALFCM, and ALFCL in experiment 2. We conclude cows choose ALFC 70.6% of the time when compared with other pellet formulations. These results suggest animals prefer pellets containing a mixture of alfalfa and corn and that flavoring agents may not enhance preference for these ingredients when included in pellet formulation.

Since early adoption of automated milk systems (**AMS**; Jacobs and Siegford, 2012), the AMS sector has grown from 8,000 units in 2009 (de Koning, 2010) to approximately 50,000 AMS worldwide (Maculan and Lopes, 2016). Two designs are available for AMS including guided traffic and free flow, of which approximately 78% of AMS farms within the upper Midwest use free-flow barn design (Siewert et al., 2019). A free-flow barn design is unique as it attempts to incentivize the animals to visit the milking robot via the offering of feed reward. This reward is usually a concentrate grain mixture in either mash or pellet form, and highly palatable ingredients such as molasses are often included (Rodenburg and Wheeler, 2002). The practice of feeding highly valued feed rewards is often employed because the amount of pellet allowance has not been observed to improve number of visits to the AMS (Halachmi et al., 2005; Bach et al., 2007; Henriksen et al., 2018). Preference by cattle for different pellet formulations can be measured and tested. A recent study evaluating different pellets showed that cattle possess a high degree of preference for pellets containing corn gluten feed, whereas preference was lowest for a pellet containing predominantly corn and wheat middlings (Carroll et al., 2023). Overall, there is a need for greater understanding of the influence of the traditional ingredients used in pellet formulations on feed preference and to determine if flavoring agents can be used to enhance preference. The objective of this experiment was to examine the differences in feed preference of 4 different pellet formulation strategies, and then examine if flavoring agents could enhance the preference of a highly preferred pellet. Our hypothesis was that a pellet formulated similar to that of a grain mix would be most preferred, and this preference would be improved with the addition of a flavoring.

Before conducting the experiment, procedures using animals were approved by the University of Nebraska–Lincoln Institutional Animal Care and Use Committee. Eight multiparous lactating Jersey cattle (100 ± 7.1 DIM, 30.5 ± 4 0.06 kg of milk yield, 18.8 ± 2.52 kg of DMI) were used for experiment 1 and 2. Each experiment was conducted with sequential elimination (Erickson et al., 2004), which is a design we have previously employed and is described by Carroll et al. (2023). Using the same design, a second experiment was carried out 115 d after the first experiment. This experiment used the same animals (215 ± 7.1 DIM, 27.6 ± 3.98 kg of milk yield, 19.6 ± 3.03 kg of DMI). This study was not conducted in an AMS, rather cows were housed in tiestalls with continuous access to water and fed once daily at 1000 h targeting 5% refusals. The sequential elimination experiment can be described as follows: after milking at 700 h and returning from an exercise pen at 930 h, cows were offered 0.50 kg of each pellet treatment. These feeds were individually offered in 30.5 × 40.6 cm plastic tubs that were placed in front of each cow. These were offered for 60 min or until a single treatment was fully consumed. The experimental phase was 9 d in total. More specifically, all 4 pelleted treatment feeds were offered in an initial feeding period from d 1 to 4. The treatment consumed in greatest amounts was deemed the most preferred feed and once identified after 4 d was no longer offered in subsequent days whereas the remaining 3 were offered for 3 d. Then, this process was repeated for 2 d. The number of days a feed was offered was according to the methods of Erickson et al. (2004). Treatments were ranked sequentially with 1 being most preferred and 4 being least. In experiment 1, preference of 4 treatment feeds were as follows: 45.7% alfalfa meal, 45.7% corn grain, and 8.57% wheat middlings (**ALFC**); 72.3% corn grain, 18.5% wheat middlings, and 9.25% dried distillers grains and solubles (**ENG**); a pellet containing 100% dehydrated alfalfa meal (**DALF**); and a pellet containing a mixture of concentrate ingredients (**GMIX**; 43.1% corn grain, 26.3% dried distillers grains and solubles, 13.8% wheat middlings 7.10% dry molasses, 3.18% soybean meal, 0.93% corn oil, and 5.6% minor constituents). Pellets were created using a 4 mm die size, and 5% water was added to each mix on an as-fed basis during pelleting. All treatments were produced at Cooper Specialty Feeds (Union, NE). Based on these results, a second experiment was conducted to observe if flavoring agents would improve animal preference. Similar to experiment 1, 4 treatment feeds were compared and these included the most preferred from the first experiment (ALFC; 45.7% alfalfa meal, 45.7% corn grain, and 8.57% wheat middlings). The remaining 3 treatments were created by adding different flavoring agents to this pellet. These included pellets were 45.7% alfalfa meal, 45.7% corn grain, 6.76% wheat middlings, and 1.81% oregano leaf (**ALFCO**), 45.7% alfalfa meal, 45.7% corn grain, 8.22% wheat middlings, 0.10% melon flavoring (Lucta, Montornès del Vallès, Barcelona, Spain) and 0.25% of a bitterness taste suppressor (BitterOff, Lucta, Montornès del Vallès; **ALFCM**), 45.7% alfalfa meal, 45.7% corn grain, 8.47% wheat middlings, and 0.10% licorice flavoring (Lucta, Montornès del Vallès; **ALFCL**).

The chemical composition of each pellet was determined. To do so, pellets were first ground through a 1-mm screen (Wiley Mill, Arthur A. Thomas Co., Philadelphia, PA) and then analyzed for DM (AOAC International, 2000), N (Leco FP-528 N Combustion Analyzer, Leco Corp., St. Joseph, MI, NDF with sodium sulfite, and α-amylase corrected for ash contamination (aNDFom; Van Soest et al., 1991), starch (Hall, 2009), ash (AOAC International, 2000), and total fatty acids (Sukhija and Palmquist, 1988) by Cumberland Valley Analytical Services Inc. (Waynesboro, PA). Pellet hardness was determined according to Berends et al. (2018), where pellet hardness was measured as the compression force (kg) required to fragment a pellet into small particles. During this time pellet diameter and length were determined with a digital Vernier caliper that measured to ±0.1 mm. Treatment rankings were analyzed for mean and SE using the PROC MEANS function of SAS (9.4; SAS Institute Inc.). Data were also analyzed with the Plackett-Luce model in R (version 1.3.1093; https://www.r-project.org/) to estimate the probability an examined pellet would be chosen first based on the rankings from the current data set. A Z-test was conducted in R on the probabilities to determine if the probability differed from the probability of no preference at 25% (1/4 × 100; Fligner and Verducci, 1988). Significance was declared with a *P*-value ≤0.05.

The aim of the first experiment was to identify differences in feed preference of pellets with different formulations. The second experiment was then conducted to determine if flavoring agents could be used to further enhance the most preferred pellet. Table 1 lists formulations, chemical composition, and pellet hardness of the 4 pellets used for experiment 1 and experiment 2. Table 2 lists results from experiment 1 as the mean and standard error of the animal preference ranking for each pellet. Pellets with means closest to 1 were considered the most preferred, whereas those closest to 4 were the least. Table 2 also provides the probability an animal would choose a given pellet differed from the null hypothesis of no preference where μ was equal to 25% (1/4 × 100; Fligner and Verducci, 1988). Table 3 presents the data from experiment 2 in a manner that follows the outline previously described in Table 2. Within the current experiment, animals were provided pellets for 4, 3, and 2 d, respectively. This sequence is outlined in Erickson et al. (2004), yet further research should further explore the effects of time on behaviors. As expected, and as a result of different formulations, the 4 pellets in experiment 1 differed in chemical composition. Within the current experiment, the high starch energy (ENG) pellet contained the greatest concentration of starch (50.3%), followed by corn and alfalfa mixture (ALFC), grain mix pellet (GMIX), and dehydrated alfalfa meal (DALF; 37.8%, 35.9%, and 1.60%, respectively); this was a function of the proportion of corn grain within the pellet. The ash content was greatest in the DALF pellet (9.30%) relative to GMIX (6.95%), ALFC (4.25%), and ENG pellet (2.86%). Pellet hardness presented in Table 1 is expressed as the kilograms of force required a pellet to crumble and create fines (Berends et al., 2018). This metric is important because it may play a role in feed preference as animals prefer to consume pellets over meals (Krogstad et al., 2021). Ash content within pellets have been previously thought to reduce pellet hardness (Abedi and Dalai, 2017). However, pellet hardness characteristics may be multifaceted and the use of ash content as a metric for pellet hardness may be an oversimplification and the number of pellet ingredients should be also considered. Within the current experiment, DALF pellet had the greatest content of ash, but the hardest pellet (41.2 ± 4.81 kg). The GMIX pellet contained 13 ingredients and a hardness of 11.9 ± 5.47 kg of force, ALFC and ENG pellet with 3 ingredients (20.6 ± 5.68 and 19.8 ± 4.24 kg of force), and DALF meal a singular ingredient (41.2 ± 4.81 kg of force). Therefore, the source of the ash added to the pellet may have a greater influence on pellet hardness relative to the total ash content itself.

**Table 1:** Ingredient inclusion and chemical composition of experimental pellets (% of pellet DM unless otherwise stated)^1^

Item	Experiment 1^2^				Experiment 2^3^			
	ALFC	ENG	DALF	GMIX^4^	ALFC	ALFCO	ALFCM	ALFCL
Ingredient								
Alfalfa meal	45.7		100		45.7	45.7	45.7	45.7
Corn, rolled	45.7	72.3		43.1	45.7	45.7	45.7	45.7
Dried corn distillers grains and solubles		9.25		26.3				
Wheat middlings	8.57	18.5		13.8	8.57	6.76	8.22	8.47
Soybean meal				3.18				
Dry molasses				7.10				
Oregano								
Corn oil				0.93				
Oregano leaf 5						1.81		
Melon flavoring^6^							0.10	
BitterOff 6							0.25	
Licorice flavoring^6^								0.10
Chemical composition, % of DM								
DM, % as-is	86.1	88.1	92.3	88.1	90.6	91.0	91.6	92.3
CP	13.3	12.9	17.9	16.1	14.4	14.2	14.5	14.4
aNDFom^7^	23.1	21.0	44.4	20.1	27.2	28.2	28.1	28.6
Starch	37.8	50.3	1.6	35.9	33.5	33.5	31.1	32.4
Total fatty acids	2.15	4.99	1.24	4.22	2.45	2.57	2.38	2.42
Ash	4.25	2.86	9.30	6.95	5.39	4.01	5.07	4.88
Hardness,^8^ kg of force (SD)	20.6	19.8	41.2	11.9	12.0	14.0	15.0	11.9
	(5.68)	(4.24)	(4.81)	(5.47)	(2.98)	(2.46)	(4.26)	(2.09)
Diameter, mm (SD)	3.96	3.93	3.98	4.06	4.08	4.06	4.05	4.07
	(0.070)	(0.134)	(0.063)	(0.171)	(0.103)	(0.052)	(0.053)	(0.082)
Length, mm (SD)	15.7	14.7	15.2	14.7	14.4	13.7	15.0	14.0
	(1.38)	(1.22)	(1.56)	(1.37)	(0.96)	(0.90)	(1.16)	(1.16)

1 Pellet formulations: ALFC = equal proportions alfalfa meal and corn grain; ENG = high corn grain pellet; DALF = dehydrated alfalfa meal; GMIX = grain mix pellet containing vitamin and mineral; ALFCO = equal proportions alfalfa meal and corn grain plus oregano leaf; ALFCM = equal proportions alfalfa meal and corn grain plus melon and a bitterness taste suppressor; ALFCL = equal proportions alfalfa meal and corn grain plus licorice flavoring.

2 Diet composition (% of diet DM): corn silage 38.5%, alfalfa hay 14.1%, fine ground corn 16.0%, expellers soybean meal 2.82%, soybean meal 9.40%, corn distillers dried grains with solubles 10.3%, rumen-protected methionine 0.11%, rumen-protected lysine 0.41%, beet molasses 1.23%, fat supplement 1.87%, ground soybean hulls 1.79%, salt 0.48%, sodium bicarbonate 0.63%, calcium carbonate 1.39%, magnesium oxide 0.26%, dicalcium phosphate 0.56%, vitamin premix 0.04%, and trace mineral premix 0.04%.

3 Diet composition (% of diet DM): corn silage 35.5%, alfalfa hay 23.6%, fine ground corn 14.2%, corn dried distillers grains 7.96%, expellers soybean meal 2.50%, soybean meal 5.01%, blood meal 1.48%, rumen-protected methionine 0.12%, rumen-protected lysine 0.23%, fat supplement 2.70%, ground soybean hulls 4.14%, salt 0.36%, calcium sulfate 0.14%, sodium bicarbonate 0.45%, calcium carbonate 1.10%, magnesium oxide 0.23%, dicalcium phosphate 0.23%, potassium chloride 0.01%, vitamin premix 0.04%, and trace mineral premix 0.04%.

4 Remaining mix (% of diet DM) = 0.09% vitamin premix formulated to supply approximately 1,133.79 kIU/d vitamin A, 181.41 kIU/d vitamin D, and 53.51 IU/d vitamin E in total rations, 0.06% trace mineral premix formulated to supply approximately 2,000 mg/kg Co, 20,000 mg/kg Cu, 2,000 mg/kg I, 5 mg/kg Fe, 100,000 mg/kg Mn, 625 mg/kg Se, and 15 mg/kg Zn in total rations, 1.30% calcium carbonate, 0.49% magnesium oxide, 1.88% sodium sesquicarbonate, 0.92% whey permeate, and 0.86% salt, white.

5 High Quality Organics, Reno, NV.

6 Lucta, Montornès del Vallès, Barcelona, Spain.

7 Amylase-treated NDF on an organic matter basis.

8 Pellet hardness was determined as the compression force (kg) required to fragment a pellet into small particles. Pellet hardness is on a DM basis.

**Table 2:** Preference scores of 4 different pelleting strategies in lactating Jersey cattle and the probability animals will choose a given pelleting strategy first based on the preference of lactating Jersey cattle from experiment 1

Item	Treatment^1^			
	ALFC	ENG	GMIX	DALF
Mean	1.38	2.13	2.88	3.13
SEM^2^	0.164	0.327	0.375	0.350
μ^3^	70.6	16.5	5.5	7.48
SE^4^	0.549	0.455	0.475	0.455
Z^5^	3.11	−0.54	−1.07	−2.55
*P*^6^	<0.01	0.59	0.28	0.01

1 Rank of pellet formulations fed at 0.5 kg per treatment, with 1 as most preferred and 4 as least preferred. ALFC = equal proportions alfalfa meal and corn grain; ENG = high corn grain pellet; DALF = dehydrated alfalfa meal; GMIX = grain mix pellet containing vitamin and mineral.

2 SEM = standard error for mean choice of each pellet.

3 μ = the estimated percentage chance a diet will be chosen when all treatments are presented.

4 SE = standard error for the percentage chance a treatment will be chosen first.

5 Z = the test to determine if the percentage chance of a treatment being chosen first by the animal is different from the percentage of no choice at 25%.

6 *P* = indicates that the preference values differ from the percentage chance of no choice at 25%.

**Table 3:** Preference scores of 4 different pelleting strategies in lactating Jersey cattle and the probability animals will choose a given pelleting strategy first based on the preference of lactating Jersey cattle from experiment 2

Item	Treatment^1^			
	ALFC	ALFCO	ALFCM	ALFCL
Mean	1.25	2.38	2.63	3.25
SEM^2^	0.164	0.263	0.375	0.164
μ^3^	81.9	8.49	6.5	3.12
SE^4^	0.648	0.462	0.481	0.491
Z^5^	3.11	−0.54	−1.07	−2.55
*P*^6^	<0.01	0.59	0.28	0.01

1 Rank of pellet formulations fed at 0.5 kg per treatment, with 1 as most preferred and 4 as least preferred. ALFC = equal proportions alfalfa meal and corn grain; ALFCO = equal proportions alfalfa meal and corn grain plus oregano leaf; ALFCM = equal proportions alfalfa meal and corn grain plus melon and a bitterness taste suppressor; ALFCL = equal proportions alfalfa meal and corn grain plus licorice flavoring.

2 SEM = standard error for mean choice of each pellet.

3 μ = the estimated percentage chance a diet will be chosen when all treatments are presented.

4 SE = standard error for the percentage chance a treatment will be chosen first.

5 Z = the test to determine if the percentage chance of a treatment being chosen first by the animal is different from the percentage of no choice at 25%.

6 *P* = indicates that the preference values differ from the percentage chance of no choice at 25%.

The observed preference ranking listed from greatest to least was (1) ALFCO, (2) ENG, (3) GMIX, and (4) DALF, and the mean (± SE) choice for each of these pellets was 1.38 ± 0.164, 2.13 ± 0.327, 2.88 ± 0.375, and 3.13 ± 0.350, respectively (Table 2). Based on these rankings, we conclude the pellet containing a mixture of ALFC pellet was the most preferred with a probability of first choice at 70.6 ± 0.549% (Table 2). It is possible that preference was greatest because animals were most familiar with these ingredients. Physical pellet characteristics like hardness may not have an overriding influence on preference of pellets. More specifically, the ingredients themselves and proportion of inclusion concentrations also contribute to the preference for the alfalfa and corn mixture pellet. Alfalfa leaf and stem and corn grain each contain unique volatile compounds contributing to a fresh herbaceous scent (Dalai et al., 2006; Xu et al., 2015), and fatty and floral scent (Zhang et al., 2022), which when combined in an even ratio, may mimic that found within the TMR the animals consumed. The cognitive system of cattle is believed to integrate the odor of a feed along with the taste (Provenza et al., 1992). These senses may have contributed to the animal's familiarity with the pellet ingredients and thus increased preference. The probability of ENG and GMIX pellets being chosen first was observed to be 16.5 ± 0.455% for the ENG and 7.48 ± 0.455% for the GMIX pellet. However, this did not differ from the mean values of 25%. The second highest preference was observed for the ENG pellet and this was surprising as the pellet formulation had been previously observed to be associated with low preference (Carroll et al., 2023). We speculate that compared with the GMIX pellet, the higher preference for ENG pellet was at least in part a result of the fact that pellet hardness was lower for the grain mix pellet (11.9 vs. 19.8 ± 5.47 kg of force). This characteristic can result in greater crumbling that leads to decreased ability for cattle to physically gather and consume pellets. It is known that the act of prehension with the tongue moves along the horizontal plane and that smaller particles are harder to capture and consume (Hudson and Frank, 1987). The least preferred pellet in our study was the pellet composed completely of dehydrated alfalfa meal as indicated by a significantly lower probability of choice of 5.50 ± 0.475%. Previous research has indicated that forages such as grass type pellets are less preferred relative to pellets containing grains such as barley or oats (Madsen et al., 2010). However, the reason for aversion to alfalfa meal is not completely clear especially since the most preferred pellet contained a small proportion of dehydrated alfalfa meal.

The highest preference for alfalfa and corn mixture pellet was maintained, and this was indicated with a preference ranking of 1.25 ± 0.164, 2.38 ± 0.263, 2.63 ± 0.375, and 3.25 ± 0.164 for ALFC, ALFCO, ALFCM, and ALFCL, respectively (Table 3). While it is possible that this is in response to the fact that the ratio of the ingredients was similar to that in TMR as previously discussed, we recognize that within the second experiment there could have been an aspect of familiarity with this pellet as animals had previously been exposed to this pellet. No difference (*P* ≥ 0.28) was observed in the probability of choice between ALFCO (8.49 ± 0.462%) and ALFCM (6.50 ± 0.481%). Oregano has been previously observed to improve palatability of hydrolyzed feather meal, which is considered be poorly palatable (Buse et al., 2021). Oregano can be used to mask certain feed characteristics including bitterness (Bobiano et al., 2019). While oregano and melon with a bitterness taste suppressor may be able to mask or even possibly impede the receptors associated with unpleasant flavors, this effect may not further enhance preference when the pellet is already highly preferred. Also, of the flavoring agents tested the probability (*P* = 0.01) of an animal choosing the pellet with added licorice flavoring was the least (3.12 ± 0.491%). Ultimately, the ALFC pellet was associated with the highest probability of choice (81.9 ± 0.648%). Thus, pellet flavoring agents may be better suited to mask unpleasant flavors or odors rather than enhancing flavor of more palatable ingredients.

The aim of this experiment was to examine 4 different pelleting strategies, and 3 flavoring agents for preference in lactating Jersey cattle. Results of the first experiment indicate that animals show an increased degree of preference for pellets containing alfalfa and corn grain. Alternatively, cows appeared to exhibit the lowest preference for a pellet completely composed of dehydrated alfalfa meal. Additionally, we observed that pellet preference for the corn and alfalfa mix was not improved with the addition of flavoring agents. Understanding pellet palatability and preference has direct implications to improve pellet formulation for AMS systems. Therefore, further research should seek to improve the knowledge surrounding the hierarchy of preference for feeds when included in pellets. Further research should also seek to identify methods to enhance palatability through the use of supplementary flavorings. Thus, direct improvements in pellet preference may occur by improving the understanding of feed preference hierarchy, determining where supplementary flavors may benefit feed preference, and considering different pellet formulation strategies based on the base ingredients of the mix.
